# MicroRNA-1985 enhances the redox capability of scallop (*Patinopecten yessoensis*) in response to poly(I:C) stimulation by targeting MNK1

**DOI:** 10.3389/fimmu.2025.1556591

**Published:** 2025-05-08

**Authors:** Linghui Yu, Huiqi Deng, Shaohua Liu, Jianpin Xia, Zhenlin Hao, Donghong Yin, Yaoyao Zhan, Yaqing Chang

**Affiliations:** ^1^ College of Bioscience and Biotechnology, Shenyang Agricultural University, Shenyang, Liaoning, China; ^2^ Key Laboratory of Mariculture & Stock Enhancement in North China’s Sea, Ministry of Agriculture and Rural Affairs, Dalian Ocean University, Dalian, Liaoning, China

**Keywords:** miR-1985, mitogen-activated protein kinase-interacting kinase 1, *Patinopecten yessoensis*, oxidative stress, innate immunity

## Abstract

To clarify the microRNA (miRNA)-target gene axis is involved in response to pathogen-associated molecular pattern (PAMP)-induced oxidative stress in shellfish, the full-length cDNA of a novel *mitogen-activated protein kinase-interacting kinase* 1 (*MNK1*) homolog gene from the scallop *Patinopecten yessoensis* (*PyMNK1*) was cloned and characterized. The interaction between miR-1985 and *PyMNK1* was verified, and then the responses and possible molecular function of miR-1985, *PyMNK1*, and miR-1985/*PyMNK1* axis to poly(I:C) (a classic virus-related PAMP) stimulation in *P. yessoensis* were explored and preliminarily dissected. The results indicate: 1) The full-length cDNA of *PyMNK1* was 5354 bp, with a high level of sequence conservation across mollusks. 2) MiR-1985 bound to the 3’-UTR of *PyMNK1* and negatively regulated the expression of *PyMNK1*. 3) *Py*MNK1 may repress the relative expression of superoxide dismutase (*SOD*) by binding its promoter. 4) Both *PyMNK1* silencing and miR-1985 overexpression promoted the expression and enzymatic activity of SOD. 5) The miR-1985/*PyMNK1* axis may be involved in the response to poly(I:C) stimulation by elevating the activity of the SOD/catalase axis. To summarize, all observations from this study indicated that *P. yessoensis* may enhance its redox capability via the miR-1985/*PyMNK1*/SOD/CAT cascade and thereby alleviate PAMP-induced oxidative stress.

## Highlights

- A targeting relationship between miR-1985 and *PyMNK1* was verified.
- *Py*MNK1 regulates *superoxide dismutase* (*SOD*) transcription by promoter binding.
- Both *PyMNK1* and miR-1985 may affect the expression and enzymatic activity of SOD.
- MiR-1985 enhances the redox capability in response to poly(I:C) stimulation by targeting *PyMNK1*.
- MiR-1985/*PyMNK1* axis may regulate poly(I:C)-induced redox homeostasis alteration by targeting *SOD*.

## 1 Introduction

Pathogen-associated molecular patterns (PAMPs) are a class of molecules structurally conserved among many pathogens that elicit a host immune response when bound by germline-encoded host receptors known as pattern-recognition receptors (PRRs) (1). Upon activation by PAMPs, PRR recognition and metabolic reprogramming occurs in activated innate immune cells, which typically increases production of reactive oxygen species (ROS) (2). Although excessive ROS may help the host to eradicate pathogens to some extent, oxidative stress induced by increased ROS production may cause molecular damage, biochemical process disruptions, and cellular dysfunction in the host (3). Therefore, eliminating PAMP-induced oxidative stress as soon as possible while defending against pathogens is an important tradeoff of host innate immune regulation.

MicroRNAs (miRNAs) are a class of small endogenous non-coding RNAs with a length of 18–25 nt that are ubiquitously expressed in metazoan organisms (4). Generally, miRNAs exert their regulatory function by binding to specific sites of their target genes, leading to altered expression of these genes (5). In recent years, the regulatory role of miRNA-target gene pairs has attracted increasing attention, particularly for elucidating the mechanisms of innate immunity regulation and anti-stress responses in many organisms (6, 7). However, the extent to which miRNAs regulate innate immunity and anti-stress responses remains poorly defined, and the regulatory signaling cascades and axes remain unclear.

Shellfish is a common model species for innate immunity research (8), and a high-value seafood with notable popularity worldwide (9). Recently, several studies hypothesized that miR-1985 might be involved in the innate immune response in *Biomphalaria tenagophila* (10), *Argopecten irradians* (11), and *Pinctada fucata martensii* (12). Combined with our preliminary bioinformatics prediction data indicating that miR-1985 may target mitogen-activated protein kinase-interacting kinase 1 (*MNK1*) in *Patinopecten yessoensis*, we thus hypothesized that miR-1985 might play a role in the innate immune response in *P. yessoensis*. To address this hypothesis, we first cloned and characterized the full-length cDNA of an *MNK1* homolog from *P. yessoensis* (*PyMNK1*) and then verified the interaction between miR-1985 and *PyMNK1*. Polyinosinic–polycytidylic acid [poly(I:C)] is a synthetic analog of double-stranded RNA (dsRNA) which is a common innate immune stimulator in aquatic animals (such as *Danio rerio*, *Strongylocentrotus intermedius*, *Scylla paramamosain*, *Chlamys farreri*, *Apostichopus japonicus*, and *Mytilus galloprovincialis*) (13–18), we therefore employed poly(I:C) as a virus-mimetics to activate the innate immune system in this study, and the response of both the miR-1985/*PyMNK1* axis and its downstream targets were investigated during the stimulation process subsequently. The observations in this study enrich the information pool of miRNA-target gene pairs and their functions, and also provide new clues for elucidating the tradeoff strategy of synchronous pathogen elimination and oxidative stress attenuation during an innate immune response in a host.

## 2 Materials and methods

### 2.1 Experimental animals and cell lines

Two hundred 12-month *P. yessoensis* individuals were collected from the Dalian Lvshun Longwangtang Farm (Dalian, Liaoning Province, China). Prior to experimentation, all specimens were maintained in 1000-L laboratory circulating seawater tanks without feeding for 3 days. *P. yessoensis* individuals were fed *Chlorella vulgaris* algae twice a day *in vivo* experiments following the method of Metian et al. (19). The specimens were temporarily incubated in freshly filtered seawater maintained at 15.0°C, with a salinity of 31.21 ± 0.20, a pH of 8.10 ± 0.03, and a dissolved oxygen concentration of 7.88 mg/L. The water was changed every 2 days.

Human embryonic kidney cells transformed with SV40 large T-antigen (HEK-293T) were used as the vector for verifying the binding sites between the miRNA and its target gene, as these cells exhibit rapid growth, robustness, and strong bioluminescent signals (20). The maintenance of HEK-293T cells (Sangon Biotechnology; Shanghai, China) followed the method of Liu et al. (21).

### 2.2 Sample collection

Nine healthy adult *P. yessoensis* were starved for 3 days. Tissues (gills, mantles, hepatopancreas, hemolymph, gonads, muscles) were then carefully collected on ice. The collection of hepatopancreases followed the method of Lyu et al. (22). To collect hemolymph, lymphocytes were centrifuged at 3000 rpm for 10 min at 4°C. All samples were immediately frozen in liquid nitrogen and stored at −80°C for subsequent total RNA extraction, genomic DNA extraction, and expression analysis.

### 2.3 Full length cDNA cloning of *PyMNK1*

Total RNA extraction, the determination of RNA integrity and concentration, synthesis of the first-strand cDNA, and rapid amplification of cDNA ends (RACE) were performed according to Ren et al. (23). The obtained cDNA was diluted and stored at −20°C. A SMARTer^®^ RACE 5’/3’ kit (Sangon Biotechnology, China) was used to synthesize a 5’/3’ terminal RACE cDNA template via qualified total RNA synthesis. The reaction and PCR procedure were performed according to the manufacturer’s instructions. The primers used for full length cDNA cloning are listed in **Table 1**. PCR products were recovered and purified using a SanPrep column DNA gel extraction kit (Sangon Biotechnology, China). The ligation, transformation, and colony PCR experiments were performed according to Ren et al. (23). After colony PCR screening, three positive clones were selected and sent to Sangon Biotechnology (Shanghai, China) for sequencing.

**Table 1:** Primers and synthetic sequences used in this study.

Primer	Primer sequence(5’ - 3’ )	Application
*MNK1*-F1	TACGCTGGAATAGCAGGTAGAGCA	ORF cloning
*MNK1*-R1	AAGTTTCTCCTACGCCTAGCGAGT	
*MNK1*-F2	TCATTGGGCGTAGATCCCGAG	
*MNK1*-R2	TGGAATGGCACTTGTGTTCTGCT	
*MNK1* 5’	AGAAGCATAGGCACCACTCCCCAGC	5’ RACE
*MNK1* 3’-F1	TTCAACCTTTGTGGAGTCGCTACA	3’ cloning
*MNK1* 3’-R1	CTACTCTATTGAAGCCGCCGAAAC	
*MNK1* 3’-F2	CGGCGGCTTCAATAGAGTAGTC	
*MNK1* 3’-R2	AAGGCTTGGTTGGAGGTATGTT	
*MNK1* 3’	CCAACCAAGCCTTCAAAGCAAGCTGCT	3’ RACE
*MNK1*-F	GATCTGGAATGCTTCGCTGGAGAG	qRT-PCR
*MNK1*-R	TCCTCGTCTTCCTGGCTGTCTTC	
*SOD* promotor-F1	TTTCTTGTCGTATTTTCGTGTCGTC	promotor cloning
*SOD* promotor-R1	GAAACAACACTGGGCTAAGGGAATACAC	
*SOD* promotor-F2	GTAATAGTTTTGAACAATGCCACACGCT	
*SOD* promotor-R2	AACCAGAAATCAAAGAGAAAGTGGCGTC	
*SOD*-F	CCAACCAAGACTCGCCTCTTACAG	qRT-PCR
*SOD*-R	CCAGTCAACAAGGTTCCACCAGTC	
*β-actin*-F	CAAACAGCAGCCTCCTCGTCAT	qRT-PCR Reference
*β-actin*-R	CTGGGCACCTGAACCTTTCGTT	
miR-1985	CCGCTGCCATTTTTATCAGTCACTGTG	qRT-PCR
RNAU6B	ACGCAAATTCGTGAAGCGTT	qRT-PCR Reference
si*MNK1*-F	GGACCCGAUUAUGGAAGAUTT	RNA interference
si*MNK1*-R	AUCUUCCAUAAUCGGGUCCTT	
si*MNK1* NC-F	UUCUCCGAACGUGUCACGUTT	
si*MNK1* NC*-*R	ACGUGACACGUUCGGAGAATT	
miR-1985 agomir	CAGUGACUGAUAAAAAUGGCAUU	miR-1985 overexpression
miR-1985 agomir Negative control	UUGUACUACACAAAAGUACUG	
miR-1985 antagomir	CACAGUGACUGAUAAAAAUGGCA	miR-1985 inhibition
miR-1985 antagomir Negative control	CAGUACUUUUGUGUAGUACAAC	

### 2.4 Superoxide dismutase promoter cloning

Genomic DNA was extracted from gills of *P. yessoensis* using a DNeasy blood and tissue kit (Qiagen, Germany), following the method of Yoon et al. (24). The DNA sequences for the *SOD* promoter were cloned based on the National Center for Biotechnology Information database (GenBank No.: 110461622). Primer 5.0 software was used to design primers for sequence amplification of the *SOD* gene promoter (**Table 1**). The ligation, transformation, and colony PCR experiments were performed according to Ren et al. (23). All primer synthesis and sequencing were completed by Sangon Biotechnology (Shanghai, China).

### 2.5 Characterization of *PyMNK1* and the *SOD* promoter sequence

Analyses to characterize *MNK1* were performed following the methods of Liu et al. (21), including complete full-length cDNA sequence assembly and annotation, sequence structure analyses, physicochemical property predictions, multiple sequence alignment, and phylogenetic analysis. The information used for the multiple sequence alignment, characteristic domain comparison, and phylogenetic analysis is shown in **Supplementary Table S1**.

A potential transcription start site of the *SOD* gene was predicted by Promoter 2.0 (https://services.healthtech.dtu.dk/services/Promoter-2.0/). An analysis of the CpG islands in the *SOD* gene promoter sequence was performed by Methprimer (http://urogene.org/cgi-bin/methprimer/methprimer.cgi) using the parameters length > 100 bp, Obs/Exp > 0.6, and GC% > 50%.

### 2.6 Quantitative real-time reverse transcription-polymerase chain reaction and western blotting

The relative expression of miR-1985, *PyMNK1*, and *SOD* was analyzed using a LightCycler^®^ 96 real-time quantitative PCR instrument (Roche Life Science, Germany). The primers used for qRT-PCR are listed in **Table 1**. *β-actin* and *RNAU6B* (*U6*) were used as internal references because they are commonly used in relative transcriptional expression analyses of aquatic animals (25). The relative expression levels of miR-1985, *PyMNK1*, and *SOD* were analyzed by the 2^−ΔΔCt^ method (25).

The recombinant protein expression of *Py*MNK1, polyclonal antibody preparation, and antibody specificity testing were completed at Mai-si Biotechnology (Wuhan, China). Total protein concentration of each sample was determined following the method of Liu et al. (21). Detailed experimental protocol of western blotting was described in the **Supplementary File 1**.

### 2.7 Binding site predictions and verification of the miR-1985/*PyMNK1* axis and *Py*MNK1/*SOD* promoter

The target site of miR-1985 in *PyMNK1* was identified by using RNA22 v2 microRNA target detection (https://cm.jefferson.edu/rna22/Interactive/) and artificial sequence alignment (Sangon Biotechnology; Shanghai, China). Plasmids expressing the wild-type (WT) *PyMNK1* containing a putative target of miR-1985 on the 3’- untranslated region (3’-UTR) and *PyMNK1* with a mutated 3’-UTR were synthesized by Sangon Biotechnology (Shanghai, China) (**Table 1**). The target fragment was inserted between the *XhoI* and *NotI* restriction sites of a luciferase plasmid, and the clones were further confirmed by sequencing. In a transfection experiment, HEK-293T cells were inoculated in 96-well white TC plates with a total volume of 100 μL. Then, two solutions were prepared: the first consisted of DMEM (10 µL); either the pmirGLO luciferase plasmid (0.16 µg), or the wild-type *PyMNK1* plasmid, or the mutant-type (MT) *PyMNK1* 3’-UTR plasmid (200 ng); and the miRNA/negative control (NC; 5 pmol). The second solution consisted of 10 μL of DMEM and 0.3 μL of Lipofectamine 2000 (Thermo Fisher Scientific, USA), and was incubated at room temperature for 5 min. Then, 25 μL of the first and 25 μL of the second solutions were combined and incubated at room temperature for 20 min. Then, 100 μL of the medium was added to each well of the 96-well white TC plate containing HEK-293T cells. After transfection (48 h), the cells were collected, and the activity was determined using a dual luciferase reporter assay system (E1980, Promega Biotechnology, Beijing). The luciferase activity was determined by calculating the signal ratio between *Renilla* luciferase and firefly luciferase using a SpectraMax i3x enzyme marker (Mei Ya Molecular Instruments, China). All experiments were performed with three replicates.

The binding sites of the promoters of *SOD* and *PyMNK1* were identified by using JASPAR (http://jaspar.genereg.net/) and artificial sequence alignment (Sangon Biotechnology; Shanghai, China). Plasmids expressing the wild-type promoter of *SOD* containing the putative target of *Py*MNK1 and wild-type *PyMNK1* were synthesized by Sangon Biotechnology (Shanghai, China). The target fragment was inserted between the *XhoI* and *NotI* sites of the luciferase plasmid, and the clones were further confirmed by sequencing. In a transfection experiment, HEK-293T cells were incubated in 96-well white TC plates with a total volume of 100 μL. Two solutions were prepared: the first consisted of 10 µL of DMEM, 0.16 µg of the pmirGLO luciferase plasmid or 200 ng of the wild-type *SOD* plasmids or 200 ng of the wild-type *Py*MNK1 and *SOD* plasmids, and 5 pmol of the pGL3-control plasmid. Preparation of the second solution and luciferase activity determination are the same as binding site verification of the miR-1985/*PyMNK1* axis.

### 2.8 Functional analysis of miR-1985 and *PyMNK1 in vivo*

The miR-1985 miRNA agomir, antagomir, and NC were designed and synthesized at Gene-Pharma (Shanghai, China; **Table 1**) and dissolved in RNase-free water to obtain a working solution of 20 nM. Then, 10 μL of the miR-1985 agomir (or antagomir) or NC (or NC inhibitor) was mixed with 10 μL of Lipofectamine 2000 and 80 μL of phosphate buffered saline (PBS) to serve as the transfection solution. Healthy *P. yessoensis* were injected with 100 μL of transfection solution or the NC solution mixture. At 24 h post transfection, the gills from each group were collected, immediately frozen in liquid nitrogen, and stored at − 80°C for further analyses, including qRT-PCR, western blotting, SOD and catalase (CAT) enzyme activity assays, and ROS content determination.

Specific small interfering RNAs (siRNAs) targeting *PyMNK1* (si*MNK1*) and RNA interference negative controls (NC-RNAi) were designed and synthesized by Sangon Biotech (Shanghai, China; **Table 1**). For *in vivo PyMNK1* knockdown, 10 μL of si*MNK1* (20 nM) or NC-RNAi was mixed with 10 μL of Lipofectamine 2000 and 80 μL of PBS to serve as the transfection solution. Healthy *P. yessoensis* were injected with 100 μL of the si*MNK1* mixture or the NC-RNAi mixture (as a control). The physiological status of all examined *P. yessoensis* individuals should be the same before and after injection to avoid the stress reaction caused by injection. At 24 h post transfection, the gills from the si*MNK1* group and the control group were collected, immediately frozen in liquid nitrogen, and stored at −80°C for further analyses, including qRT-PCR, western blotting, SOD and CAT enzyme activity assay, and ROS content determination.

### 2.9 Poly(I:C) stimulation and sample collection

A total of 60 healthy *P. yessoensis* were selected and injected intramuscularly with sterile seawater containing 50 μL of poly(I:C) (InvivoGen, USA; 1.0 mg/mL in sterile seawater) following the method of Yang et al. (26). The injection operation of the *P. yessoensis* was shown in **Supplementary Figure S1**. Three individuals from each group were randomly selected at 0 (as control), 6, 12, 24, 48, and 72 h post stimulation (hps) (n = 3). The gills of each individual in each group were collected, immediately frozen in liquid nitrogen, and stored at −80°C for further analyses, including qRT-PCR, western blotting, SOD and CAT enzyme activity assay, and ROS content determination.

### 2.10 ROS content and enzyme activity determination

Prior to ROS content and enzyme activity analyses, gill samples were homogenized and the protein concentration of the gill homogenates was determined followed the method of Liu et al. (21). ROS content determination was carried out by using a commercial kit (Beyotime Biotechnology, Shanghai, China) following the method of Hao et al. (27). Enzyme activity analyses of SOD and CAT were carried out using commercial kits (BC5160, Solarbio Science & Technology Co., Ltd, Beijing, China; S0038, Beyotime Biotechnology, Shanghai, China) following the method of Yu et al. (28) and Zhang et al. (29).

### 2.11 Data analysis

All data were expressed as mean ± S.D. Prism 9 (GraphPad, USA) was used for data processing, data collation, and chart drawing. Statistical analysis was performed using independent samples *t*-tests and one-way analysis of variance (ANOVA) in Prism 9. Significant differences and extremely significant differences were set at *P* < 0.05 and *P* < 0.01, respectively.

## 3 Results

### 3.1 *PyMNK1* cDNA sequence characterization

**Figure 1:**
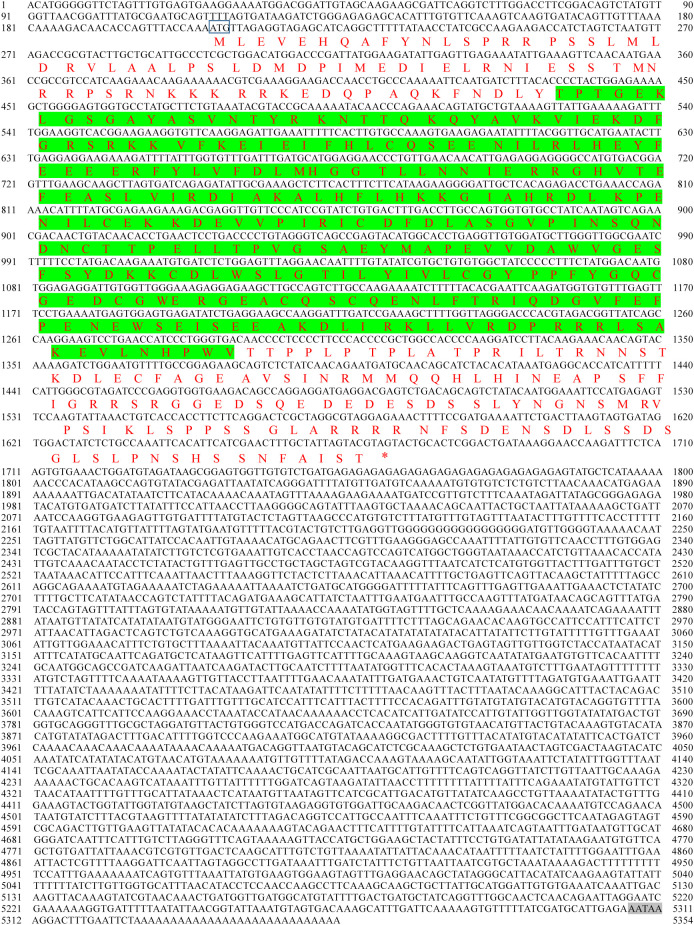
Nucleotide and predicted amino acid sequences of *PyMNK1*. The numbers on the left alternate from top to bottom as the nucleotide sequence and the deduced amino acid sequence numbers; the black font indicates the nucleotide sequence, and the red font indicates the amino acid sequence. The domain is shaded in green. The start codon (ATG) is framed by a blue box; the asterisk (*) represents the termination codon (TGA), and the polyadenylation signal (AATAA) is framed by a box.

**Figure 2:**
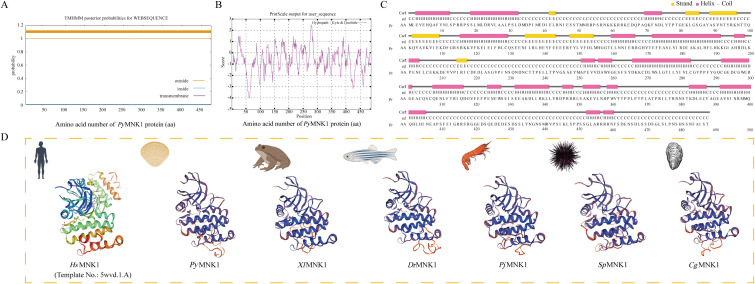
Sequence characteristic analysis of *Py*MNK1. **(A)** Transmembrane structure analysis of *Py*MNK1 protein. **(B)** Hydrophobic or hydrophilic analysis of *Py*MNK1 protein. **(C)** Predicted protein secondary structure of *Py*MNK1 protein. Yellow, pink and grey highlight strands, helices and coils, respectively. **(D)** Predicted 3D structure of the *Py*MNK1 protein and MNK1 proteins from five other species. The 3D structure of *Homo sapiens* MNK1 protein (*Hs*MNK1; Swiss-model No.: 5wvd.1.A) was used as the template. *Py*MNK1, *Xl*MNK1, *Dr*MNK1, *Pj*MNK1, *Sp*MNK1, *Cg*MNK1 represent MNK1 proteins from *Patinopecten yessoensis, Xenopus laevis*, *Danio rerio*, *Penaeus japonicus*, *Strongylocentrotus purpuratus*, *Crassostrea gigas*, respectively.

As shown in the results, the cDNA of *PyMNK1* (GenBank accession No. PP737163) is 5354 bp, including a 265-bp 5’-UTR, a 1407-bp open reading frame, and a 3682-bp 3’-UTR (**Figure 1**). The open reading frame of *PyMNK1* encoded a 488 aa protein with a predicted molecular weight of 53.40 kDa and theoretical pI of 5.56. Further analyses indicated that the *Py*MNK1 protein was a non-transmembrane hydrophilic protein (**Figures 2A, B**). A structure prediction analysis determined that the secondary structure of the *Py*MNK1 protein contained 22 α-helices, 7 β-strands, and 30 random coils (**Figure 2C**). A characteristic domain analysis revealed that the *Py*MNK1 protein contained one S_TKc domain (PAS-A: 285 aa, residues 54–371). The *Py*MNK1 protein for *Homo sapiens* (SWISS-MODEL No.: 5wvd.1.A) was used as the template for the 3D structure prediction of the *Py*MNK1 protein from *P. yessoensis* and *Py*MNK1 proteins from five other species. Compared with the template, 3D structure similarities of the MNK1 proteins from the six examined species were 86.75% (*Xenopus tropicalis*), 82.51% (*Danio rerio*), 66.10% (*Penaeus japonicus*), 64.75% (*Strongylocentrotus purpuratus*), 60.81% (*Crassostrea gigas*), and 58.76% (*P. yessoensis*) (**Figure 2D**). This indicated that the 3D structures of MNK1 homologs were relatively conserved from invertebrates to higher vertebrates, and that they may perform similar functions.

**Figure 3:**
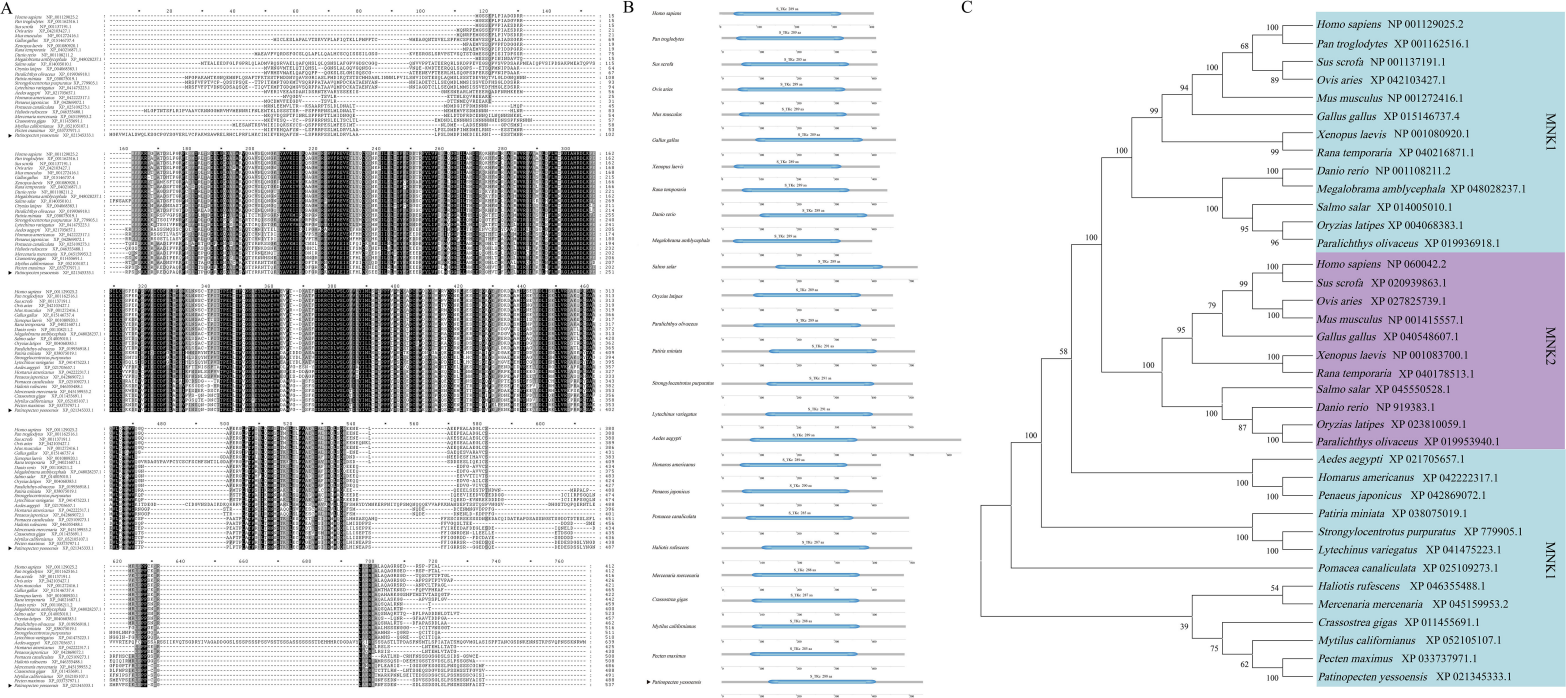
Phylogenetic analysis of *Py*MNK1. **(A)** Multiple sequence alignment of MNK1 proteins from *Patinopecten yessoensis* and 25 other species. **(B)** Schematic diagram of conserved domains of MNK1 homologs from *P. yessoensis* and 25 other species. **(C)** Neighbor-joining phylogenetic tree of MNK1 homologs from *P. yessoensis* and 36 other species. *Py*MNK1 is marked with a “◂”; the numbers at the tree nodes indicate bootstrap support values based on 1,000 replicates.

A multiple sequence alignment analysis found that the average similarity between the *Py*MNK1 sequence and MNK1 sequences from 25 other species was 36.98% (**Figure 3A**). The highest similarity (86.78%) was observed between *Py*MNK1 and an MNK1 homolog from *Pecten maximus*. A characteristic domain comparison indicated that the *Py*MNK1 and MNK1 proteins from 25 other eukaryotic organisms shared relatively high homologies in the S_Tkc domain (75.25%). Moreover, the number and types of characteristic domains of *Py*MNK1 were the same as those of the MNK1 proteins from the 25 other eukaryotic organisms (**Figure 3B**). A phylogenetic analysis showed that the *Py*MNK1 protein and the MNK1 protein from *P. maximus* were clustered into one branch and belonged to the clade of mollusk, which was consistent with traditional taxonomy (**Figure 3C**).

### 3.2 *SOD* promoter sequence characterization and interaction with *Py*MNK1

**Figure 4:**
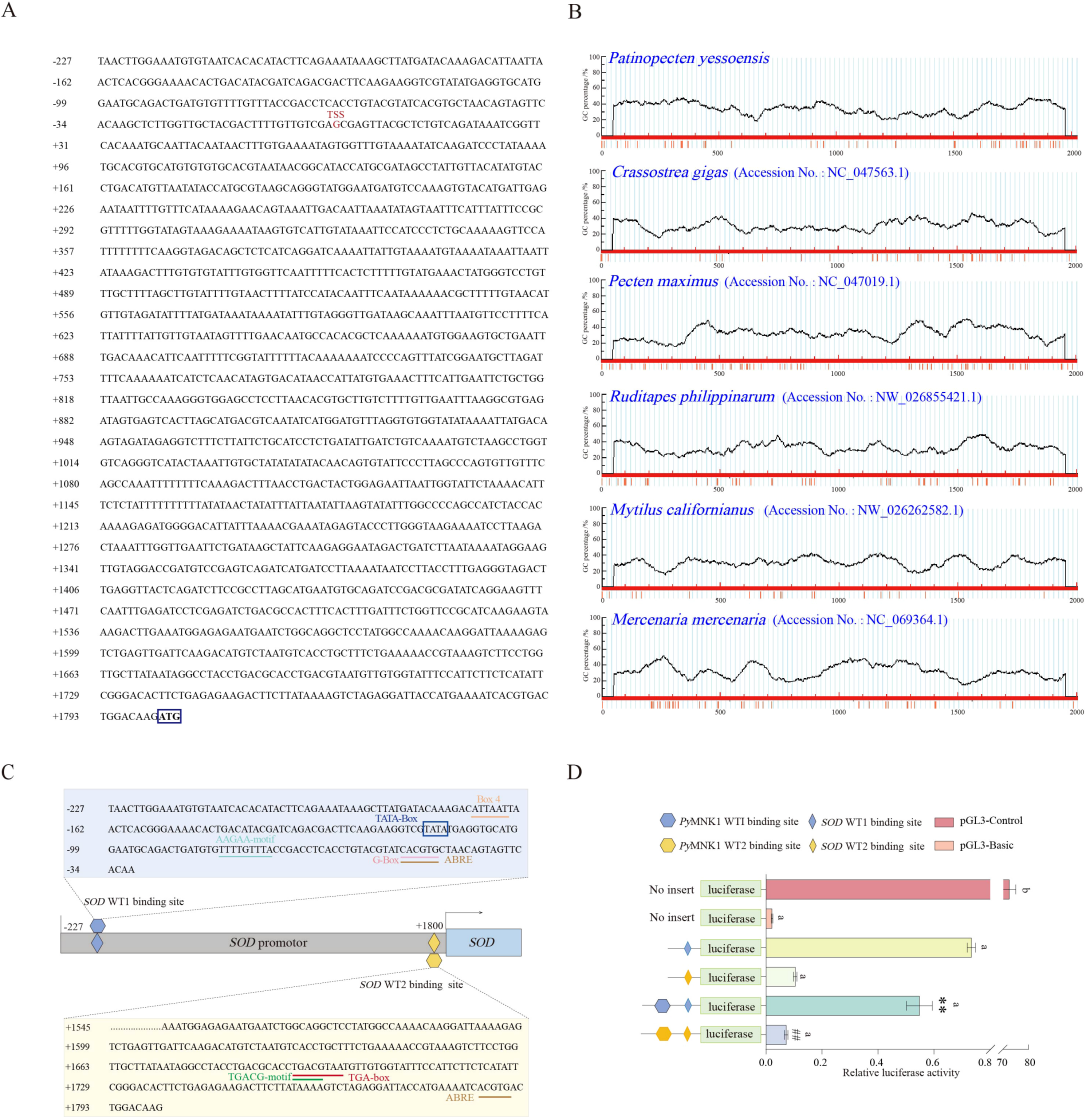
Sequence and CpG island predictions of the *SOD* promotor, binding site identification, and verification of the *PyMNK1* and *SOD* promotor. **(A)** The sequence of the *SOD* promoter. The transcription initiation site (TSS) is in the red font. The blue box represents the start codon (ATG). **(B)** CpG island prediction of the *SOD* promotor from *Patinopecten yessoensis* and five other species. **(C)** Schematic representation of putative *Py*MNK1 targeting sites and regulatory elements in the *SOD* promotor. **(D)** An analysis of relative luciferase activity. Different lower-case letters (e.g., a, b) represent the significance levels between different groups (*P* < 0.05). ** represent *P* < 0.01 *vs*. *SOD* WT1 binding sites group. ## represent *P* < 0.01 *vs*. *SOD* WT2 binding sites group. pGL3-basic, promoter vector; pGL3-control, positive control; WT, wild type.

The promoter of the *SOD* gene is approximately 2027 bp in length, and the predicted transcription start site was the guanine (G; labeled as +1 bp) located 1800 bp upstream of the translation initiation site (ATG) of the *SOD* gene (**Figure 4A**). The online software MethPrimer prediction results did not identify any CpG islands in the *SOD* gene promoter sequence, a result which was similar to five other species (*C. gigas*, *P. japonicus*, *Mercenaria mercenaria*, *Mytilus californianus*, and *Ruditapes philippinarum*) (**Figure 4B**). There were 10 consecutive T nucleotides located at position +1151 to +1160 bp in the *SOD* promoter of *P. yessoensis*. In addition, five core promoter regulatory elements were identified in *SOD* WT1 binding sites: an Abscisic acid responsive element (ABRE) (−47 bp), G-Box (−47 bp), AAGAA motif (−74 bp), TATA box (−111 bp), and Box 4 (−165 bp) (**Figure 4C**). In addition to these five core promoter transcription elements, three promoter basic regulatory elements were identified in *SOD* WT2 binding sites: a TGACG motif (+1692 bp), TGA box (+1692 bp), and ABRE (+1786 bp) (**Figure 4C**).

To verify the binding sites of *Py*MNK1 on the *SOD* promoter, luciferase reporter vectors containing WT fragments of *Py*MNK1 and *SOD* were constructed (**Figure 4C**). The results showed that the WT1 *SOD* group and WT2 *SOD* group had remarkably higher luciferase activity than the NC group (**Figure 4D**), indicating that the cloned *SOD* gene promoter sequence bound to transcription factors to initiate transcription of the *SOD* gene. The results showed that the WT1 *Py*MNK1 + WT1 *SOD* group and WT2 *Py*MNK1 + WT2 *SOD* group had remarkably lower luciferase activity than the WT1 *SOD* group and WT2 *SOD* group, respectively (**Figure 4D**), indicating the presence of a binding site between *Py*MNK1 and miR-1985. These results indicated that *Py*MNK1 can negatively regulate the promoter of the *SOD* gene and affect its transcriptional activity.

### 3.3 Spatial expression and interaction analyses of miR-1985 and *PyMNK1*

**Figure 5:**
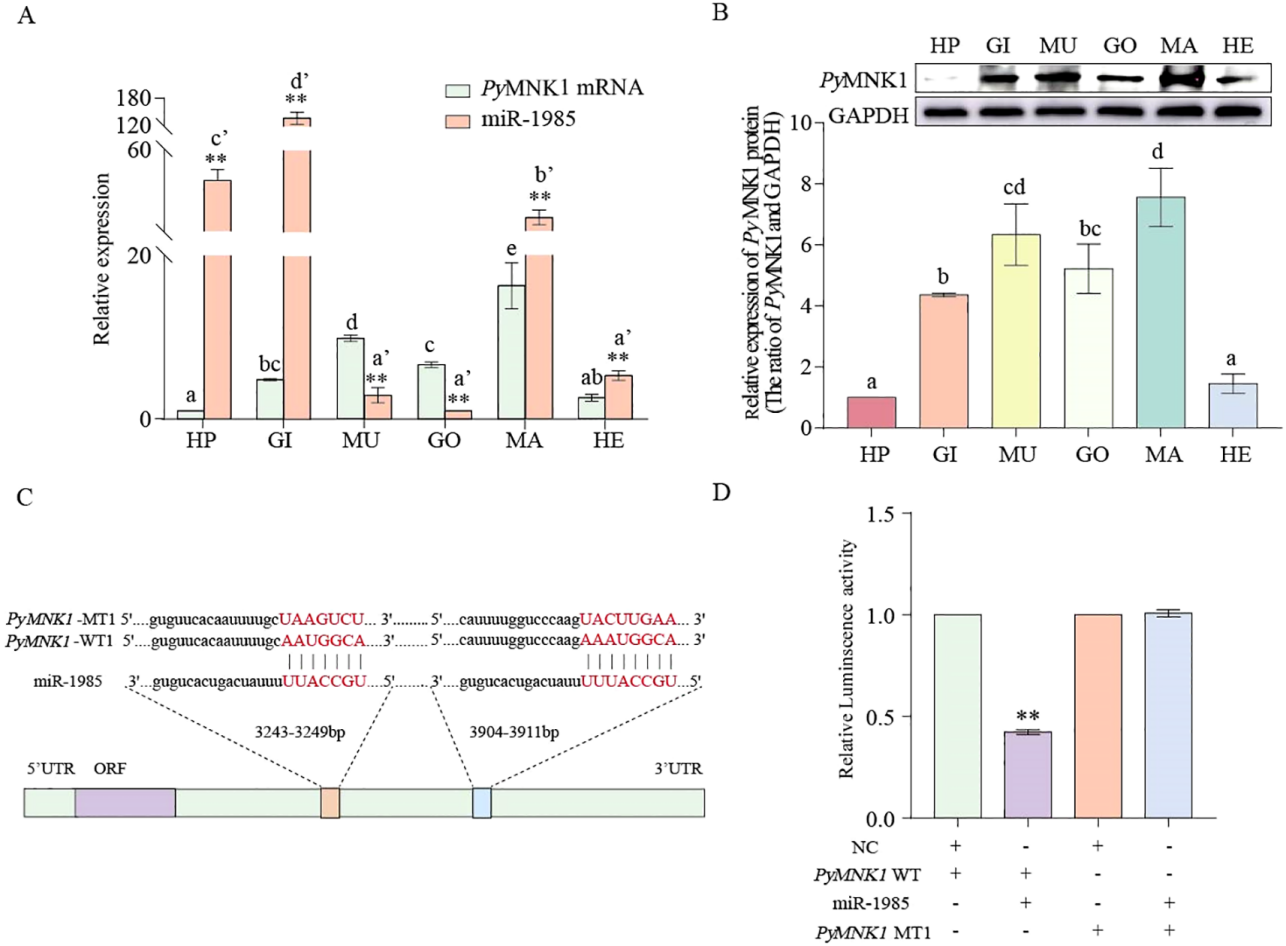
Spatial expression pattern, binding site identification, and verification of miR-1985 and *PyMNK1*. **(A)** Spatial expression pattern of miR1985 and *PyMNK1.* mRNA. HP: hepatopancreas; GO: gonads; MU: muscles; GI: gills; MA: mantles; HE: hemolymph. **(B)** Spatial expression pattern of *Py*MNK1 protein. **(C, D)** Schematic representation of the putative miR-1985 targeting site in *PyMNK1* mRNA and an analysis of relative luciferase activity. Different lower-case letters (e.g., a or a’) indicate significant differences between different tissues (*P* < 0.05). “**” represents an extremely significant difference between miR-1985 and *Py*MNK1 mRNA in the same tissue or between NC + *PyMNK1*-WT and miR-1985 + *PyMNK1*-WT (*P* < 0.01).

The results of qRT-PCR showed that both miR-1985 and *PyMNK1* mRNA were expressed in all the examined tissues. The relative expression of *PyMNK1* mRNA in the mantle was the highest of all the examined tissues. In contrast, miR-1985 was highly expressed in gills, followed by the hepatopancreas (**Figure 5A**). An overall opposite expression trend of miR-1985 and *PyMNK1* mRNA was observed across all examined tissues. Specifically, the relative expression of miR-1985 was higher than that of *PyMNK1* mRNA in hepatopancreas, gills, mantles, and hemolymph, whereas, lower than that of *PyMNK1* mRNA in muscles and gonads. Western blotting results showed that the *Py*MNK1 protein was expressed in all the examined tissues, with the relative expression level from low to high according to the following: hepatopancreas < hemolymph < gills < gonads < muscles < mantles. This was similar to the pattern of *PyMNK1* mRNA expression (**Figure 5B**).

**Figure 6:**
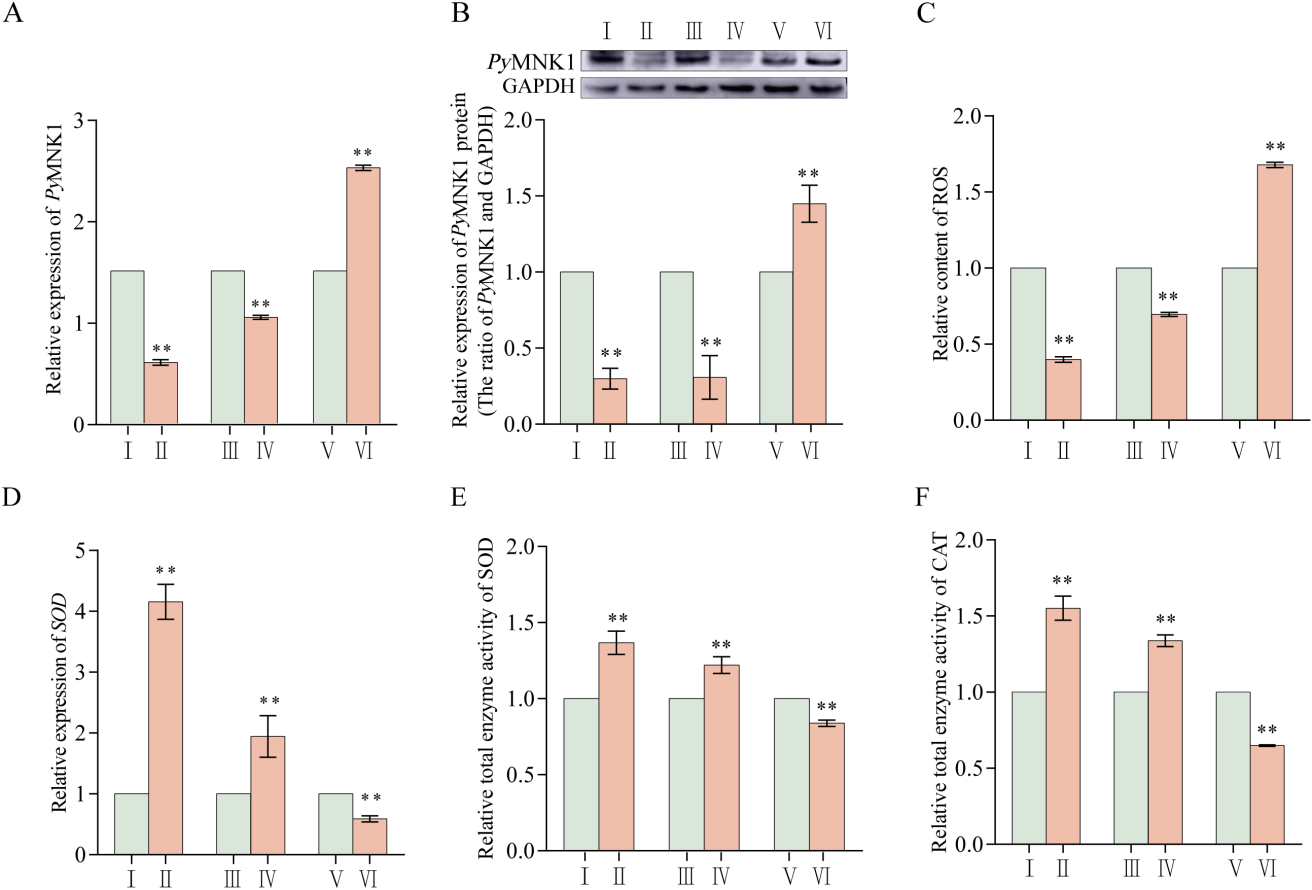
Effects of *PyMNK1* and miR-1985 on mRNA expression, ROS content, and total SOD and CAT enzymatic activity changes in gills of *Patinopecten yessoensis*. **(A, B)** Effects of *PyMNK1* and miR-1985 on *PyMNK1* mRNA and protein expression. **(C)** Effects of *PyMNK1* and miR-1985 on ROS content. **(D-F)** Effects of *PyMNK1* and miR-1985 on *SOD* mRNA expression and enzymatic activity of SOD and CAT. I: negative control of *PyMNK1* silencing group (NC-RNAi); II: *PyMNK1* silencing group (RNAi); III: negative control of miR-1985 overexpression group (NC-Agomir); IV: miR-1985 overexpression group (Agomir); V: negative control of miR-1985 inhibition group (NC-Antagomir); VI: miR-1985 inhibition group (Antagomir). * represents *P* < 0.05; ** represents *P* < 0.01.

To verify the binding sites of miR-1985 on *PyMNK1*, luciferase reporter vectors containing WT and MT fragments of the *PyMNK1* 3’-UTR were constructed (**Figure 5C**). The results showed that the WT 3’-UTR + miR-1985 mimic group had remarkably lower luciferase activity than the NC group (**Figure 5D**), indicating the presence of a binding site between *PyMNK1* and miR-1985. Further *in vivo* functional verification experiments revealed that the relative expression levels of *Py*MNK1 (mRNA and protein) decreased significantly when miR-1985 was overexpressed (*P* < 0.01), whereas the relative expression of *Py*MNK1 (mRNA and protein) increased significantly when the expression of miR-1985 was inhibited (*P* < 0.01), similar to the results of the RNAi experiment for *PyMNK1* (**Figures 6A, B**). These findings indicated that miR-1985 negatively regulated *Py*MNK1 protein expression post-transcriptionally, and that *PyMNK1* siRNA inhibited *PyMNK1* expression at the transcriptional level.

### 3.4 Effects of miR-1985 and *PyMNK1* on mRNA expression and enzymatic activity of SOD in *P. yessoensis*

The relative expression (mRNA and protein) of *Py*MNK1 significantly decreased after *PyMNK1* silencing and miR-1985 overexpression, indicating that both RNAi and miR-1985 overexpression significantly inhibited the relative expression of *PyMNK1* (**Figures 6A, B**). At the same time, the relative content of ROS after *PyMNK1* silencing or miR-1985 overexpression was significantly reduced (**Figure 6C**). Moreover, after *PyMNK1* silencing or miR-1985 overexpression, the relative expression of *SOD* mRNA significantly increased and the enzymatic activity of SOD and CAT also significantly increased (**Figures 6D–F**).

### 3.5 Changes in ROS, miR-1985, *Py*MNK1 expression (mRNA and protein), and SOD and CAT activity after poly(I:C) stimulation

**Figure 7:**
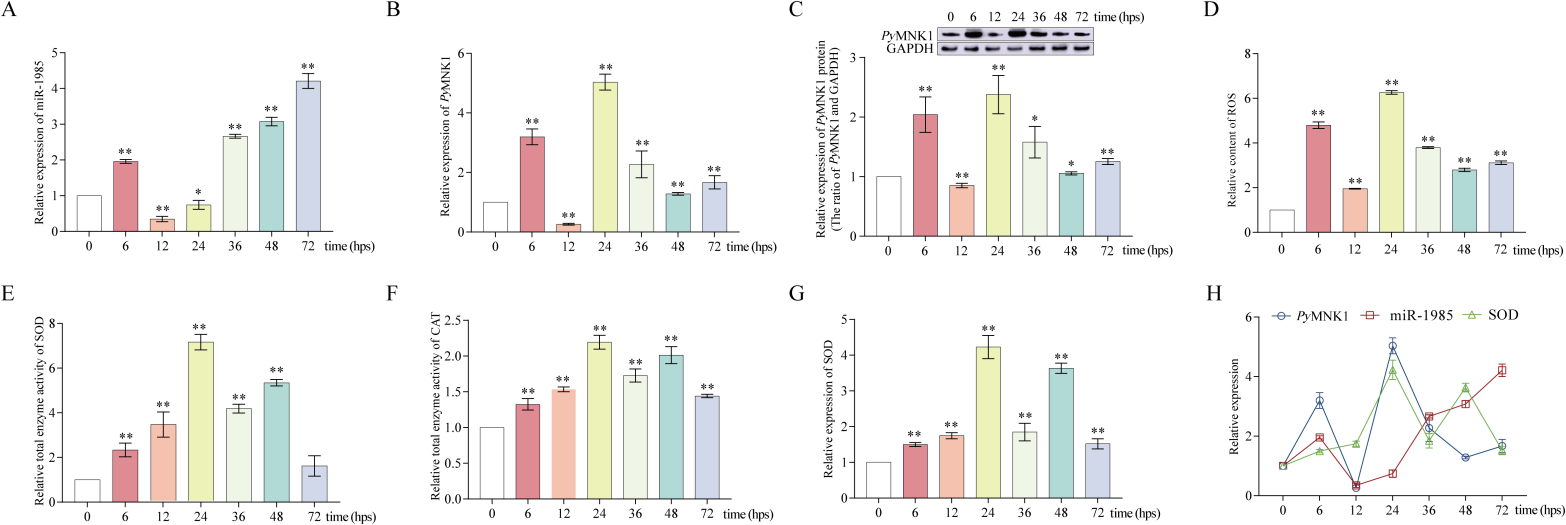
Poly(I:C) stimulation altered miR-1985, *Py*MNK1 (mRNA and protein), and *SOD* mRNA expression, ROS content, and total SOD and CAT enzymatic activity in gills of *Patinopecten yessoensis*. **(A-C)** Relative expression of miR-1985 and *Py*MNK1 (mRNA and protein) in gills of *P. yessoensis* after poly(I:C) stimulation. **(D–F)** Changes in the ROS content and total SOD and CAT enzymatic activity in gills of *P. yessoensis* after poly(I:C) stimulation. **(G)** Relative expression of *SOD* mRNA in gills of *P. yessoensis* after poly(I:C) stimulation. **(H)** Relative expression of miR-1985, *PyMNK1* mRNA, and *SOD* mRNA after poly(I:C) stimulation. * represents *P* < 0.05 vs. 0 hps; ** represent *P* < 0.01 vs. 0 hps.

After *P. yessoensis* individuals were stimulated with poly(I:C), the relative expression level of miR-1985 in gills was upregulated at 6 hps, downregulated at 12 hps, and then upregulated from 36 to 72 hps (**Figure 7A**). The relative expression of *Py*MNK1 mRNA in gills of *P. yessoensis* initially increased, then decreased, then increased again, then decreased, and finally increased after poly(I:C) stimulation. The three peaks of mRNA expression appeared at 6, 24, and 72 hps (**Figure 7B**). The trend of the relative expression of *Py*MNK1 protein in gills of *P. yessoensis* after poly(I:C) stimulation was similar to the trend of mRNA expression (**Figure 7C**).

The relative ROS content exhibited an overall increasing trend after poly(I:C) stimulation with three peaks appearing at 6, 24, and 72 hps (**Figure 7D**), consistent with the relative expression trend of MNK1 mRNA and protein. Although the relative enzyme activity of both SOD and CAT in *P. yessoensis* gills exhibited an generally increasing trend after poly(I:C) stimulation, the alteration trend was opposite to that of the relative ROS content at 6–12 and 36–72 hps (**Figures 7E, F**). In addition, the relative expression trend of SOD mRNA was consistent with the alteration change of SOD relative enzyme activity (**Figures 7G, H**).

### 3.6 Effects of miR-1985 on the SOD/CAT axis by regulating *PyMNK1* in gills after poly(I:C) stimulation

**Figure 8:**
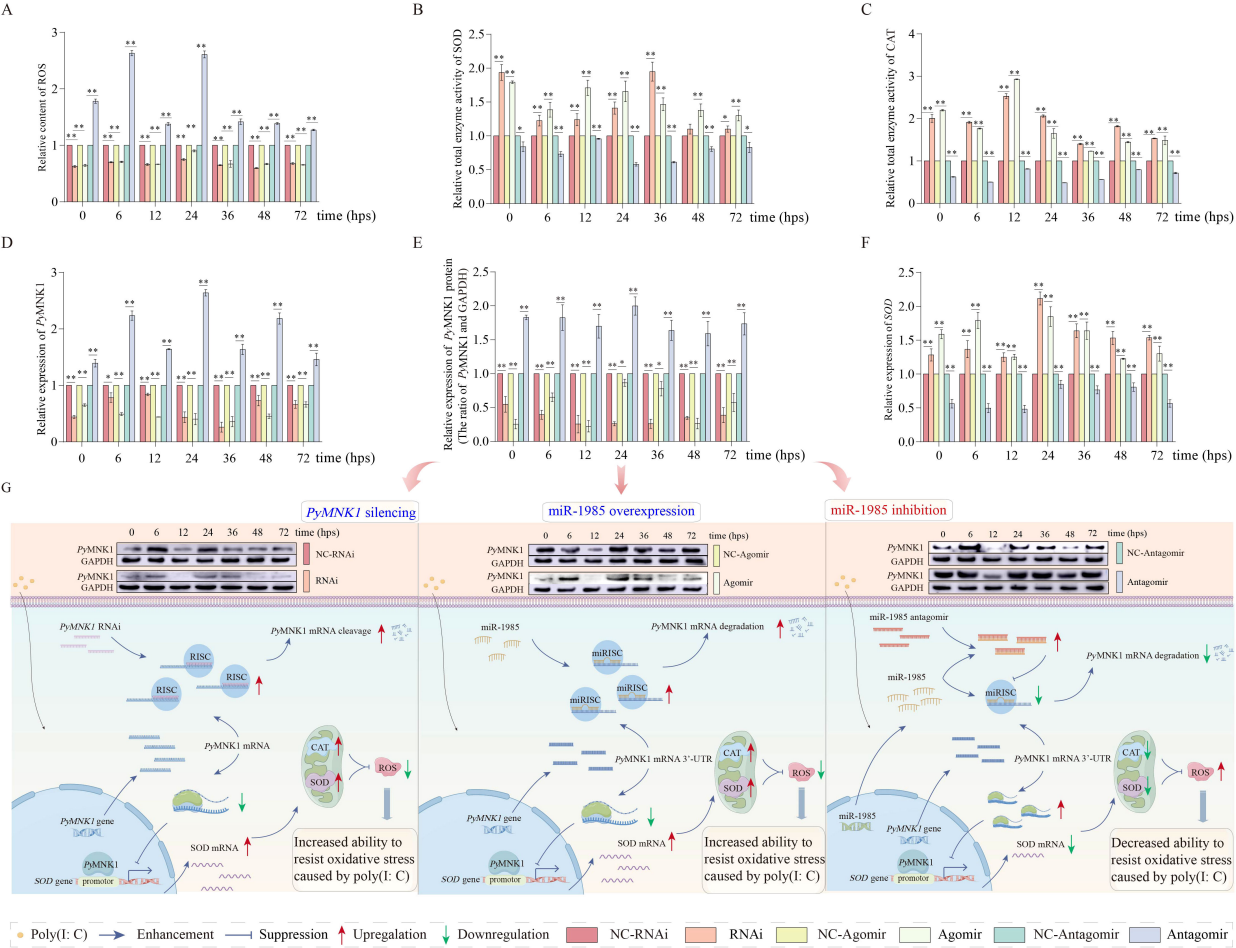
Effects of miR-1985 expression and *PyMNK1* silencing on *PyMNK1* (mRNA and protein) expression, ROS content, and total enzymatic activity of SOD and CAT in gills of *Patinopecten yessoensis* after poly(I:C) stimulation. **(A–C)** Effects of miR-1985 expression and *PyMNK1* silencing on ROS content, total SOD and CAT enzyme activity in gills of *P. yessoensis* after poly(I:C) stimulation. **(D–E)** Effects of miR-1985 expression and *PyMNK1* silencing on *Py*MNK1 (mRNA and protein) expression in gills of *P. yessoensis* after poly(I:C) stimulation. **(F)** Effects of miR-1985 expression and *PyMNK1* silencing on *SOD* mRNA expression in gills of *P. yessoensis* after poly(I:C) stimulation. **(G)** Schematic diagrams of miR-1985 and *PyMNK1* involved in responding to poly(I:C) stimulation in *P. yessoensis*. Schematic images were drawn by Figdraw (http://www.figdraw.com). NC-RNAi, negative control of *PyMNK1* silencing group; RNAi, *PyMNK1* silencing group; NC-Agomir, negative control of miR-1985 overexpression group; Agomir, miR-1985 overexpression group; NC-Antagomir, negative control of miR-1985 inhibition group; Antagomir, miR-1985 inhibition group. * represents *P* < 0.05; ** represents *P* < 0.01.

Because the trend of the ROS content was consistent with the trend of MNK1 expression, we further investigated whether miR-1985 downregulated ROS production through the SOD/CAT axis by regulating *PyMNK1* in the gills of *P. yessoensis* after poly(I:C) stimulation (**Figures 8A–G**).

In *PyMNK1* expression inhibition (RNAi) group or miR-1985 overexpression (agomir) group, the relative ROS content decreased in the gills of *P. yessoensis* after poly(I:C) stimulation compared with that of the control group (**Figure 8A**). Moreover, the relative total SOD and CAT enzyme activity increased higher than that of the control group in the gills of *P. yessoensis* after poly(I:C) stimulation when knocking down the expression of *PyMNK1* or overexpressing miR-1985 (**Figures 8B, C**).

In the miR-1985 inhibition (antagomir) group, the relative ROS content extremely significantly increased in the gills of *P. yessoensis* after poly(I:C) stimulation (**Figure 8A**). The relative expression of *Py*MNK1 (mRNA and protein) exhibited generally increasing trends in the gills of *P. yessoensis* after poly(I:C) stimulation (**Figures 8D, E**). The relative total SOD and CAT enzyme activity exhibited generally decreasing trends in the gills of *P. yessoensis* after poly(I:C) stimulation (**Figures 8B, C**).

## 4 Discussion

Regulation of gene expression at the post-transcriptional or translational level to balance the production of ROS is increasingly being studied in the field of redox biology and innate immunology (30). In this study, we focused on clarifying the regulatory mechanism of miR-1985 on *PyMNK1* gene expression and the function of the miR1985/*PyMNK1* axis in response to PAMP-induced oxidative stress (ROS production) in shellfish.

As we expected, sequence identification data indicated that there were two binding sites for miR-1985 on the 3’-UTR of *PyMNK1* mRNA. A luciferase reporter assay confirmed that miR-1985 may attenuate *PyMNK1* expression by binding its 3’-UTR. Moreover, the trends of the relative expression trends of miR-1985 and the *PyMNK1* gene were opposite of each other, based on a spatial expression analysis. The binding sites, interaction relationship, and spatial expression pattern between miR-1985 and the *PyMNK1* gene were consistent with the conserved binding and interaction mechanism generally present between an miRNA and its target genes (31).

As epigenetic factors, miRNAs affect phenotypes by altering the function of their target genes (32). Therefore, a functional exploration of the *PyMNK1* gene then become one of the concerns of this study after confirming the existence of the targeted regulatory relationships between miR-1985 and the *PyMNK1* gene. A sequence characterization data analysis revealed that *MNK1* shares average similarities of 42.96% and 60.48% with MNK1 homologs from invertebrates and vertebrates, respectively, suggesting that MNK1 homologs share similar functions from invertebrates to vertebrates. Expression analysis data also showed that *Py*MNK1 was ubiquitously expressed in all tissues examined in this study, and showed a similar expression pattern to that of *H. sapiens* (33), suggesting a relatively conserved expression pattern of MNK1 homologs from invertebrates to vertebrates. It has been well documented that MNK1 homologs correlate closely with survival signals in response to a wide variety of stimuli (34–37). In particular, MNK1 is mainly known as a protein that promotes viral infection (37). In this study, overall upregulation of *Py*MNK1 (both mRNA and protein) was observed after virus-related PAMP [poly(I:C)] stimulation (except 12 hps) accompanied with an obvious and continuous increase of the ROS content in gills of *P. yessoensis*. This observation was consistent with the ROS production rule of viral infections (38), and also suggests that *Py*MNK1 may be involved in the immune response to viral infections in *P. yessoensis*, similar to the function of MNK1 homologs documented in mammals (37). Furthermore, similar trends of *Py*MNK1 relative expression and relative ROS content suggest a possible tight linkage between these two. Further RNAi data provided evidence that the relative ROS level decreased when the expression of *PyMNK1* was knocked down in gills of *P. yessoensis*. What is the possible mechanism of *PyMNK1*-induced ROS content changes? Commonly, the SOD/CAT axis is the most prominent redox cascade that catalyzes ROS to H_2_O and O_2_, in which SOD first catalyzes the dismutation of O_2_^−^ to hydrogen peroxide (H_2_O_2_), and CAT in turn detoxifies H_2_O_2_ to H_2_O and O_2_ (39). It is acknowledged that the stronger the enzymatic activity of the SOD/CAT axis, the better the scavenging effect of ROS (40). Typically, the activity of a given enzyme can be enhanced by increasing its concentration (41). In this study, we noticed that after knocking down the expression of *PyMNK1*, the total enzymatic activity of the SOD/CAT axis was enhanced, and *SOD* mRNA expression increased. We thus hypothesized that *Py*MNK1 may suppress the enzymatic activity of the SOD/CAT axis by reducing the SOD concentration, through targeting the transcription of the *SOD* gene. As we expected, a dual-luciferase reporter assay revealed that *Py*MNK1 directly bound to the promoter of the *SOD* gene and attenuated its transcription. It has been well documented that MNK1 homologs often bind to eukaryotic initiation factor 4E (eIF-4E) to perform their biological functions (42). Although we did not explore the role of the MNK1/eIF-4E axis in this study, our results at least confirmed that MNK1 directly bound to the promoter of the *SOD* gene, thus acting as a transcriptional suppressor. This observation also suggests that MNK1 homologs may be involved in cellular process regulation in both a direct and in-direct manner. Taken together, we conclude that inhibition of *PyMNK1* may increase the total enzymatic activity of the SOD/CAT axis by enhancing SOD transcription, consequently reducing ROS content and enhancing the immune response. Similarly, it was proposed by Xu et al. (43) that MNK1 suppression may be used as an efficient immunotherapy route to block cancer invasion and metastasis by enhancing the immune response (44). We thus speculated that the immune-regulatory function of MNK1 homologs is highly conserved from invertebrates to vertebrates.

Finally, in the context that *PyMNK1* is one target gene of miR-1985 and *PyMNK1* may affect the ROS content by regulating the SOD/CAT axis, we thus speculated that miR-1985 may affect the ROS content by targeting *PyMNK1*. As we thought, overexpression of miR-1985 inhibited the expression of *PyMNK1*, enhancing the total enzymatic activity of the SOD/CAT axis and reducing ROS content in gills of *P. yessoensis* after poly(I:C) stimulation, which was consistent with the results of *PyMNK1* RNAi (*PyMNK1* expression knock down) in this study. Moreover, we also noticed that the miR-1985/*PyMNK1* axis did function during the entire poly(I:C) stimulation period. Specifically, the expression of both miR-1985 and *PyMNK1* in gills of *P. yessoensis* fluctuated and were generally similar from 0 to 24 hps, which was inconsistent with the interaction relationship obtained in this study, suggesting that miR-1985 and *PyMNK1* may not form a functional axis during this period, and the regulatory mechanism of total enzymatic activity of the SOD/CAT axis and the ROS content require further elucidation. However, from 36 to 48 hps, opposite fluctuations of miR-1985 and *PyMNK1* expression in gills of *P. yessoensis* were observed, which was consistent with the interaction identified in this study. Moreover, the changes in the relative expression of *SOD*, total enzymatic activity of the SOD/CAT axis, and the ROS content were also consistent with the altered expression of *Py*MNK1. These observations suggest that miR-1985 may bind to *PyMNK1* to form miR-1985/*PyMNK1*, and *Py*MNK1 may in turn bind to the *SOD* promoter, and thus a cascade involving miR-1985/*Py*MNK1/SOD/CAT perform the function of poly(I:C)-induced ROS scavenging in gills of *P. yessoensis* from 36 to 48 hps. At 72 hps, changes of the relative mRNA expression of *SOD* and total enzymatic activity of the SOD/CAT axis conform to the regulatory rule of *Py*MNK1 as determined in this study, although consistent trends of miR-1985 and *PyMNK1* expression in gills of *P. yessoensis* were still observed. This observation indicated that the miR-1985/*PyMNK1* axis may disintegrate at 72 hps, although the miR-1985/*PyMNK1* axis may not regulate ROS, *Py*MNK1 may affect the total enzymatic activity of the SOD/CAT axis to achieve ROS reduction by regulating transcription of the *SOD* gene. As for the mechanism of ROS regulation at 72 hps, further investigation is required. Taken together, we conclude that miR-1985 may reduce poly(I:C)-induced ROS production by regulating the total enzymatic activity of the SOD/CAT axis by negatively targeting *PyMNK1*.

### Conclusions

In this study, we preliminarily clarified the function and mechanism of the miR-1985/*PyMNK1* axis in response to PAMP-induced oxidative stress in shellfish for the first time. The results revealed that miR-1985 may negatively regulate the expression of *PyMNK1*; furthermore, *Py*MNK1 may bind to the promoter of SOD, thus attenuating the expression of *SOD* and reducing the activity of the SOD/CAT axis. We thus conclude that shellfish may enhance their redox capability via the miR-1985/*PyMNK1*/SOD/CAT axis and thereby alleviate PAMP-induced oxidative stress. Moreover, we can also take advantage of the observations in this study to not only enhance disease prevention and control of cultured shellfish, but also to improve the molecular selective breeding of shellfish in the future. Specifically, we could design or mine miR-1985 activators or MNK1 homolog inhibitors in shellfish aquaculture, and we could also mine valuable SNPs from MNK1 homolog genes or SOD promoter sequences to develop new molecular assistive selective breeding markers for shellfish cultivation. In general, the observations from this study enrich our knowledge of miRNA/mRNA axis functions in redox homeostasis of aquatic invertebrates, and provide new clues for developing disease control strategies for economical shellfish aquaculture at the molecular level.

## Data Availability

The original contributions presented in the study are included in the article/**Supplementary Material**. Further inquiries can be directed to the corresponding authors.
