# Supplementary figures and images for: MicroRNA-1985 enhances the redox capability of scallop (Patinopecten yessoensis) in response to poly(I:C) stimulation by targeting MNK1
Source: Front Immunol. 2025 May 8;16:1556591. doi: 10.3389/fimmu.2025.1556591 (PMC12095029; doi:10.3389/fimmu.2025.1556591)

**Supplementary Figure S1 Image of injection operation of the *Patinopecten yessoensis.***


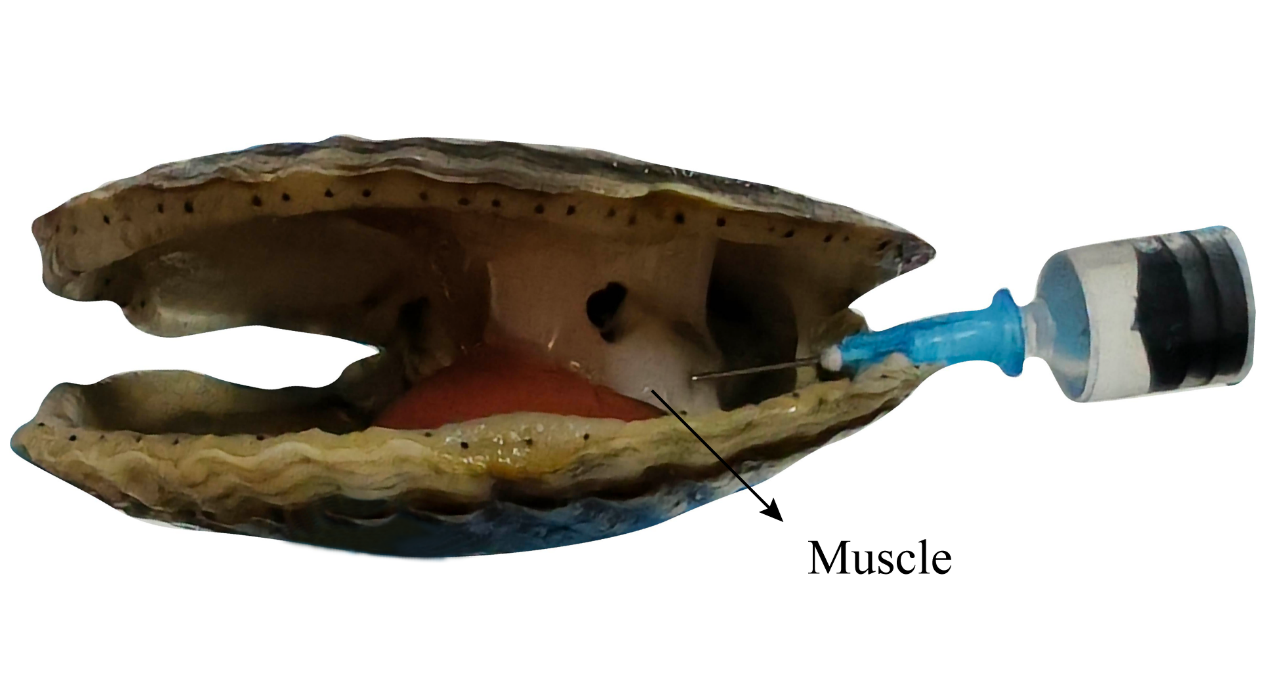

Supplement: Supplementary file 1 [file SupplementaryFile1.docx]
