# Supplementary material for: MicroRNA-1985 enhances the redox capability of scallop (Patinopecten yessoensis) in response to poly(I:C) stimulation by targeting MNK1
Source: Front Immunol. 2025 May 8;16:1556591. doi: 10.3389/fimmu.2025.1556591 (PMC12095029; doi:10.3389/fimmu.2025.1556591)
Supplement: Supplementary file 2 [file DataSheet1.docx]

**Western blotting of MNK1 and GAPDH**

Proteins were extracted from gills of *Patinopecten yessoensis* using RIPA lysis buffer (Beyotime Biotechnology Co., Ltd., Shanghai, China), separated on a 10% SDS-PAGE gel, and transferred to PVDF membranes (Roche, Switzerland). After blocking with 5% nonfat milk in TBST (10 mM Tris-HCl, pH 7.4, 150 mM NaCl, and 0.05% Tween-20) for 2 h, the membrane was incubated with a primary antibody (anti-MNK1(1:750 dilution) or GAPDH (1:5000 dilution) antibody in TBST) for 3 h. After three washes in TBST, horseradish peroxidase-labeled goat anti-rabbit IgG (Abbkine Scientific Co., Ltd., diluted 1:5000 in TBST) was used as the secondary antibody and incubated for 2 h at room temperature. Unbound IgG was then washed away, and the signals were visualized with BeyoECL Plus (Beyotime Biotechnology Co., Ltd., Shanghai, China), according to the manufacturer’s instructions. Protein bands were obtained using a ProteinSimple FluorChem E ultrasensitive, fully automated imaging analysis system (Bio-Techne, USA). The gray value of the imaged protein band was analyzed by Image J software, and the relative expression of the target protein was expressed as the ratio of the gray value of the target protein to the GAPDH gray value of the reference protein.
