# Supplementary material for: MicroRNA-1985 enhances the redox capability of scallop (Patinopecten yessoensis) in response to poly(I:C) stimulation by targeting MNK1
Source: Front Immunol. 2025 May 8;16:1556591. doi: 10.3389/fimmu.2025.1556591 (PMC12095029; doi:10.3389/fimmu.2025.1556591)
Supplement: Supplementary file 3 [file Table1.docx]

**Table S1 Amino acid sequence information for multiple sequence alignment, constructing phylogenetic tree, and drawing schematic diagram of conserved domain of MNK1 family homologs.**

| Taxon | Species | Sub-family | Accession No. |
| --- | --- | --- | --- |
| Mammals | *Homo sapiens* | MNK1 | NP_001129025.2 |
|  | *Pan troglodytes* | MNK1 | XP_001162516.1 |
|  | *Sus scrofa* | MNK1 | NP_001137191.1 |
|  | *Ovis aries* | MNK1 | XP_042103427.1 |
|  | *Mus musculus* | MNK1 | NP_001272416.1 |
|  | *Homo sapiens* | MNK2 | NP_060042.2 |
|  | *Sus scrofa* | MNK2 | XP_020939863.1 |
|  | *Ovis aries* | MNK2 | XP_027825739.1 |
|  | *Mus musculus* | MNK2 | NP_001415557.1 |
| Birds | *Gallus gallus* | MNK1 | XP_015146737.4 |
|  | *Gallus gallus* | MNK2 | XP_040548607.1 |
| Amphibians | *Xenopus laevis* | MNK1 | NP_001080920.1 |
|  | *Rana temporaria* | MNK1 | XP_040216871.1 |
|  | *Xenopus laevis* | MNK2 | NP_001083700.1 |
|  | *Rana temporaria* | MNK2 | XP_040178513.1 |
| Fish | *Danio rerio* | MNK1 | NP_001108211.2 |
|  | *Salmo salar* | MNK1 | XP_014005010.1 |
|  | *Oryzias latipes* | MNK1 | XP_004068383.1 |
|  | *Paralichthys olivaceus* | MNK1 | XP_019936918.1 |
|  | *Danio rerio* | MNK2 | NP_919383.1 |
|  | *Salmo salar* | MNK2 | XP_045550528.1 |
|  | *Oryzias latipes* | MNK2 | XP_023810059.1 |
|  | *Paralichthys olivaceus* | MNK2 | XP_019953940.1 |
| Arthropoda | *Aedes aegypti* Arthropoda | MNK1 | XP_021705657.1 |
|  | *Homarus americanus* | MNK1 | XP_042222317.1 |
|  | *Penaeus japonicus* | MNK1 | XP_042869072.1 |
| Mollusca | *Pomacea canaliculata* | MNK1 | XP_025109273.1 |
|  | *Mercenaria mercenaria* | MNK1 | XP_045159953.2 |
|  | *Crassostrea gigas* | MNK1 | XP_011455691.1 |
|  | *Mytilus californianus* | MNK1 | XP_052105107.1 |
|  | *Pecten maximus* | MNK1 | XP_033737971.1 |
|  | *Haliotis rufescens* | MNK1 | XP_046355488.1 |
| Echinodermata | *Patiria miniata* | MNK1 | XP_038075019.1 |
|  | *Patiria miniata* | MNK1 | XP_038075019.1 |
|  | *Strongylocentrotus purpuratus* | MNK1 | XP_779905.1 |
|  | *Lytechinus variegatus* | MNK1 | XP_041475223.1 |

|  |
| --- |
